# Mitochondrial Dysfunction Pathway Alterations Offer Potential Biomarkers and Therapeutic Targets for Ovarian Cancer

**DOI:** 10.1155/2022/5634724

**Published:** 2022-04-20

**Authors:** Liang Shen, Xianquan Zhan

**Affiliations:** ^1^Department of Gynecology, Shandong Provincial Hospital Affiliated to Shandong First Medical University, 324 Jingwu Weiqi Road, Jinan, Shandong 250021, China; ^2^Medical Science and Technology Innovation Center, Shandong First Medical University, 6699 Qingdao Road, Jinan, Shandong 250117, China; ^3^Shandong Key Laboratory of Radiation Oncology, Cancer Hospital of Shandong First Medical University, 440 Jiyan Road, Jinan, Shandong 250117, China

## Abstract

The mitochondrion is a very versatile organelle that participates in some important cancer-associated biological processes, including energy metabolism, oxidative stress, mitochondrial DNA (mtDNA) mutation, cell apoptosis, mitochondria-nuclear communication, dynamics, autophagy, calcium overload, immunity, and drug resistance in ovarian cancer. Multiomics studies have found that mitochondrial dysfunction, oxidative stress, and apoptosis signaling pathways act in human ovarian cancer, which demonstrates that mitochondria play critical roles in ovarian cancer. Many molecular targeted drugs have been developed against mitochondrial dysfunction pathways in ovarian cancer, including olive leaf extract, nilotinib, salinomycin, Sambucus nigra agglutinin, tigecycline, and eupatilin. This review article focuses on the underlying biological roles of mitochondrial dysfunction in ovarian cancer progression based on omics data, potential molecular relationship between mitochondrial dysfunction and oxidative stress, and future perspectives of promising biomarkers and therapeutic targets based on the mitochondrial dysfunction pathway for ovarian cancer.

## 1. Introduction

### 1.1. The Importance of Mitochondria in Cancer

The mitochondrion is the “power house” of a cell and provides more than 90% of adenosine triphosphates (ATPs) for cells. In recent years, as an important organelle in human body cells, mitochondria have been found to participate in the regulation of different physiological and pathological processes, for example, cell survival, proliferation, and migration, and function in tumor initiation, progression, and metastasis [1–3]. Mitochondria are the target and feedback center of various regulators of energy metabolism. Studies have confirmed that abnormal amino acid metabolism, fatty acid metabolism, and glucose metabolism widely occur during the progression of tumorigenesis [4]. Abnormal mitochondrial metabolism is tightly associated with the proliferation, metastasis, and survival process of cancer cells. Moreover, mitochondria are the cellular factories that produce reactive oxygen species (ROS). Accumulating evidence demonstrates that dysregulation of ROS promotes tumor malignant progression [5]. The mitochondrion, a key regulator of apoptosis, can enhance the antiapoptotic ability of cancer cells and thus cause cancer cells to rapidly proliferate [6]. Mitochondria, as the core component of intracellular calcium pool regulation, can regulate intracellular calcium homeostasis through a variety of calcium transport systems on mitochondrial membranes. Abnormalities in mitochondrial calcium homeostasis are closely related to mitochondrial dysfunction and the development of tumors [7]. Mitophagy, as selective autophagy targeting mitochondria, is one of the important regulatory mechanisms to maintain mitochondrial homeostasis [8, 9]. Mitophagy is associated with various diseases, such as cancers, neurodegenerative diseases, and immune diseases [10–12]. Thus, mitochondria are closely involved in many processes such as tumorigenesis, proliferation, invasion, and metastasis and have become a hotspot in the field of cancer research.

### 1.2. The Changes of Mitochondria in Ovarian Cancer

Ovarian cancer is the most lethal gynecologic malignant tumor in women globally [13]. Currently, the pathogenesis of ovarian cancer has not been fully clarified, and the suspected causal factors include genetics, ovulation, environmental factors, and hormones [14]. The standard treatment for ovarian cancer patients is primary debulking surgery plus platinum-based adjuvant chemotherapy. Despite the initial response rates being high after first-line treatment, most ovarian cancer patients will eventually relapse and have a poor outcome with a 5-year survival rate of only up to 46%, most of which are due to late diagnosis and a high incidence of chemoresistance [15]. Therefore, the improvement of the early-stage diagnostic rate of ovarian cancer has very important clinical significance [16]. Previous studies have found that mitochondrial dysfunctions are extensively and directly implicated in ovarian cancers [13, 17, 18]. For example, the number of mitochondrial DNAs (mtDNAs) is significantly increased in ovarian cancers relative to controls [19]. Scanning electron microscope analysis demonstrates that the loosened mitochondria in cytoplasm of ovarian cancer cells have a moth-eaten appearance with a decrease in cristae width and cristae junction diameter [20, 21]. Also, changes in mitochondrial dynamics could influence the phenotype of different subsets of ovarian cancer cells [22].

### 1.3. Study on Mitochondrial Dysfunctional Pathways in Ovarian Cancer

**Figure 1:**
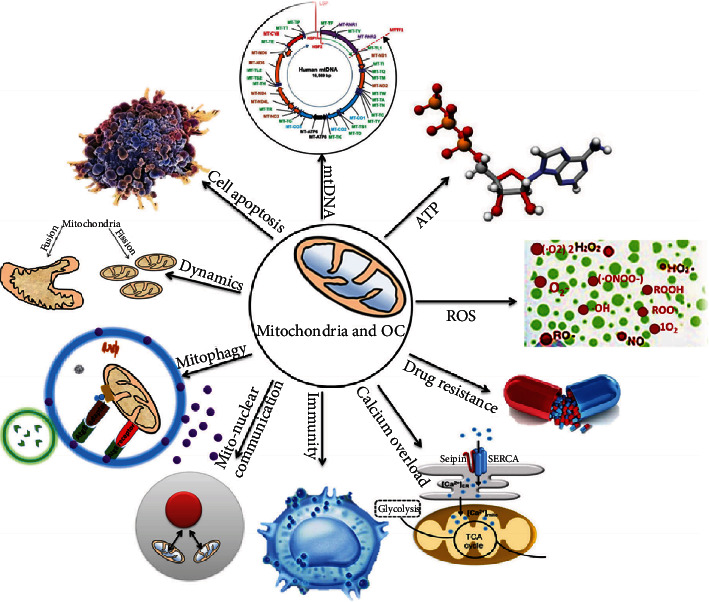
Essential roles of mitochondria in ovarian cancer. ATP: adenosine triphosphate; ROS: reactive oxygen species. Reproduced from Li and Zhan (2020) [18], with permission from John Wiley & Sons Ltd., copyright 2020.

The mitochondrion is the critical intracellular organelle that controls many important physiological and pathological processes in cancer, for example, energy metabolism, oxidative stress, mtDNA mutation, cell apoptosis, mitochondria-nuclear communication, dynamics, autophagy, calcium overload, immunity, and drug resistance [23–27] (Figure 1). The mitochondrion is the core of cellular energy metabolism, oxidative stress, and cell signaling. Altered energy metabolism is recognized as a hallmark of cancer [28]. Oxidative stress is closely associated with tumorigenesis and cancer development. Mitochondrial dysfunction has been proposed as a causal factor of ovarian cancer. The carcinogenesis and development of ovarian cancer are associated with multiparameter and multilevel pathological changes. Single-omics study commonly provides limited information for the complex molecular mechanisms, whereas multiomics studies have offered a new means to design targeted biomarkers for ovarian cancer therapy with the use of different genes, proteins, and metabolites in individual or in combination. This review article will assist in understanding mitochondrial dysfunction pathway alterations in ovarian cancer from different omics viewpoints.

## 2. Omics Study Status of Mitochondrial Dysfunction Pathways in Ovarian Cancer

The advancement of multiomics technologies (genomics, transcriptomics, proteomics, and metabolomics) and their relationship with individual phenotypes of patients are driving the medicine paradigm shift from a “one-size-fits-all” method to a predictive, preventive, and personalized medicine (3P medicine), which helps elucidate underlying mechanisms of human disease and provide reliable biomarkers and therapeutic targets for more detailed patient stratification and more effective personalized therapy. Here, we first review the general status of different omics in mitochondrial dysfunction pathway alterations in ovarian cancer and then summarize the research progress of integrative analysis of two or more types of omics data.

### 2.1. Genomic Study of Mitochondrial Dysfunction Pathways in Ovarian Cancer

The occurrence and development of cancer are a process of accumulative alterations in a genomic sequence. Mitochondria are multifunctional organelles, which control energy production, oxidative stress, metabolic signaling, and apoptotic pathways, and hence play crucial roles in tumorigenesis. The mtDNA is a circular and double-stranded DNA with 16500 nucleotides [29], which contains 37 genes that encode 13 proteins for oxidative phosphorylation, 2 rRNAs, and 22 tRNAs for protein synthesis within mitochondria, and has a noncoding control region—the displacement loop (D-loop) [30]. The mtDNA is unique because it exists multiple copies per cell, and the copy number depends upon the cell energy demand. Apart from these proteins encoded by mtDNA genes, most proteins in the mitochondrial respiratory chain are still encoded by nuclear DNA (nDNA) and then are transported into mitochondria [31]. Numerous proteins encoded by nDNA are involved in the generation of new mitochondria (mitochondrial biogenesis), and these proteins are involved in numerous mitochondrial functions such as oxidative phosphorylation (OXPHOS), apoptosis, and mtDNA replication and gene expression. Furthermore, nDNA-encoded mtDNA genes act as regulators of mitochondrial biogenesis and participate in nuclear-mitochondrial DNA cross-talks [32]. The interplay between both nuclear and mitochondrial genomes can influence the pathophysiological processes of disease including cancer.

In terms of mtDNA mutations, mutations usually occur in mtDNA genes of ovarian cancers (up to 60%), which might be the results of mitochondrial dysfunction. Apart from the D-loop region, 12S and 16S rRNA genes and cytochrome b genes are the preferred zone for mtDNA mutation in ovarian cancers [33]. Another study based on ovarian cancer samples and matched normal tissues with sequencing of mtDNA variants reveals 352 mtDNA variants [34]. Among these 352 variants, 38.3% variants are insertions and deletions, which suggests mtDNA instability in ovarian cancers, and high-frequency mutations occur at A263G, A1438G, and A8860G [34].

In terms of SNP changes, SNPs in the mtDNA and nDNA might be responsible for the pathogenesis of ovarian cancer. For example, SNPs at various nucleotide positions, including 73A/G, 207G/A, and 523C/del, are associated with increased susceptibility to ovarian cancers [35]. Another study demonstrates that three SNPs in D-loop regions (446 C/A, 324 C/G, and 309 C/T) are useful to predict postoperational survival and that two alleles at nucleotide positions 309 and 324 are independent predictors of ovarian cancer outcome [36]. A population-based study consisting of 89 patients with primary ovarian cancer found that age-at-onset in ovarian cancer women can be predicted by certain SNPs in the D-loop regions [37]. SNPs occurring at nucleotide alleles 248, 524, and 16 304 in the D-loop region are related to the age-at-onset. Patients with the minor alleles, 248 G and 16 304 T genotypes, have lower age of onset than patients with 248 A and 16 304 C, respectively. However, for the minor allele, 524C, age-at-onset of patients is higher than that of patients with a higher frequency of 524 deletions. Study finds that variants in mitochondrial biogenesis genes play a role in influencing the susceptibility of ovarian cancers. A multicenter, large-scale study of ovarian cancer demonstrates that 128 SNPs from 22 mitochondrial genetic regions along with 2839 SNPs localized at 138 nuclear-encoded genes participate in steroid hormone metabolism, mitochondrial biogenesis, and OXPHOS pathways, which is associated with ovarian cancer risk [38]. For example, CO1 (cytochrome c oxidase 1) is significantly related to ovarian cancer risk, and asynonymous SNP in CO1, T6777C appears to decrease the risk of ovarian cancer. Moreover, the study of three nDNA pathways demonstrates that 25 genes and 1051 SNPs involved in mitochondrial biogenesis are associated with ovarian cancer risk, including Nrf1 (nuclear respiratory factor 1), MTERF (mitochondrial transcription termination factor), PPARGC1A (peroxisome proliferator-activated receptor gamma, coactivator 1 alpha), CAMK2D (calcium/calmodulin-dependent protein kinase D), and ESRRA (estrogen-related receptor alpha) [38].

In terms of mtDNA content, mtDNA content in ovarian cancer cells is significantly elevated compared to the normal ovary, which meets the increased mitochondrial function need for favoring tumor growth. Quantitative analysis of the mtDNA copy number in plasma and whole blood of serious ovarian cancer patients reveals that whole-blood mtDNA copy number significantly varies between healthy controls and epithelial ovarian cancer patients [19].

Mitochondrial genetic alterations are a critical hallmark of ovarian cancer. These alterations particularly affect the electron transport chain because the OXPHOS pathway is the main pathway that is altered in ovarian cancers and other cancers. Furthermore, due to the smaller size of the mitochondrial genome, mtDNA sequencing is cost-effective and highly scalable in clinical settings. Study finds that mtDNA homoplasmic mutations occur in early preneoplastic and cancerous lesions, and the association between mtDNA copy number alterations and clinicopathological features demonstrates the importance of the mtDNA copy number as prognostic biomarkers in cancers.

### 2.2. Transcriptomics Study of Mitochondrial Dysfunction Pathways in Ovarian Cancer

Since the advent of the postgenome era, transcriptomics has been extensively used in all fields of medicine due to its high accuracy and throughput [39]. A transcriptome is a bridge to link the genome with the proteome [40]. Gene regulation is a complex network system at multiple levels, and regulation at the transcriptional level is the primary means for all organisms to control their specific gene expressions. The human transcriptome is composed of a considerable number of protein-coding mRNAs as well as noncoding RNAs [41].

In terms of mRNAs, one study based on Gene Expression Omnibus (GEO) and The Cancer Genome Atlas (TCGA) databases demonstrates the positive correlations between ovarian cancer biomarkers (such as CD44) and glycolytic biomarkers (such as hexokinase 2 (HK2), lactate dehydrogenase A (LDHA), and ENO1) [42]. LDHA is a HIF1*α*-targeted glycolytic gene, which is upregulated in ovarian cancer cells [43]. Altered energy metabolism is regarded as a hallmark of cancer. Enhanced glycolysis is a feature of cancer cells and tissues, which is commonly named as the Warburg effect [44]. Mitochondria occupy a central stage in cellular altered energy metabolism. Mitochondrial dysfunction is closely related to reprogramming of energy metabolism, which causes tumorigenesis and tumor progression. The tumor microenvironment (TME) plays crucial roles in tumor initiation and promotion of ovarian cancers, which might be the therapeutic targets. Previous studies also show that glycolysis-related gene expressions are lower in CD8+ Treg cells that coculture with SKOV3, compared to CD8+ T cells cultured alone [45]. Moreover, differentially expressed genes (DEGs) profiles are studied in peripheral CD4+ T cells from ovarian cancer patients (*n* = 5) and controls (*n* = 5), which identifies 5,175 DEGs in peripheral CD4+ T cells and finds that the metabolic pathway is the most significantly enriched pathway, and eight glycolysis-related DEGs (GLUT1, HIF1*α*, mTOR, PKM2, ENO1, GPI, PDK1, and LDH*α*) are significantly increased in CD4+ T cells from ovarian cancer peripheral blood [46].

Long noncoding RNAs (lncRNAs) and miRNAs are critical players in regulating large-scale protein-coding genes in tumor-associated pathways [47]. A study finds 39 differentially expressed miRNAs between ovarian cancer and controls, including 29 downregulated and 10 upregulated miRNAs [48]. In addition, miR-450a is the most significantly downregulated miRNAs in ovarian cancer, which restricts the metastatic capacity of ovarian cancer cells via targeting several mitochondrial mRNAs, such as translocase of inner mitochondrial membrane domain-containing protein 1 (TIMMDC1), aconitase 2 (ACO2), and ATP synthase F1 *β* subunit (ATP5B) [49]. TIMMDC1, a mitochondrial inner membrane protein, is confirmed to be associated with mitochondrial complex I [50]. ACO2 participates in the tricarboxylic acid cycle (TCA) by converting citrate to isocitrate within the TCA pathway [51]. ATP5B is one of the subunits of mitochondrial ATP synthase, which is termed as complex V to function as a mitochondrial proton pump [52]. The miR-450a can downregulate ATP5B and TIMMDC1 to impair mitochondrial functions. Moreover, miR-450a affects mitochondrial functions and the activities of ACO2, subunit 2 of NADH dehydrogenase (ND2), and TIMMDC in TCA pathways to decrease OXPHOS levels and then further increase the consumption of glucose. Therefore, many miRNAs can modulate mitochondrial functions by regulating miRNA-targeting specific genes.

Additionally, lncRNAs have acted as important regulators in several biological processes, such as gene expression, transcription, and posttranscriptional modifications [53]. lncRNA SNHG3 has been discovered to associate with ovarian cancer survival through analysis of the TCGA database. Moreover, lncRNA SNHG3 is involved in the energy metabolism process through the regulation of EIF4AIII and miRNAs, which could bind to PKM (pyruvate kinase) in glycolysis, PDHB (pyruvate dehydrogenase E1 component subunit *β*) and IDH2 (isocitrate dehydrogenase 1) in TCA cycle, and ubiquinol-cytochrome c reductase hinge protein (UQCRH) in OXPHOS pathways [54]. It clearly demonstrates that lncRNA SNHG3 is closely associated with cellular energy metabolism and controls a series of important metabolic pathways in ovarian cancers.

These findings demonstrate miRNA and lncRNA function as a critical layer of the regulation at the transcriptional level and also indicate the functional diversity and complexity of energy metabolism in the mitochondrial dysfunction pathway system.

### 2.3. Proteomics Study of Mitochondrial Dysfunction Pathways in Ovarian Cancer

Proteomics is an effective methodology that can analyze the entire proteins in cells, tissues, and organisms, including protein subtypes, posttranslational modifications, protein interactions, and protein structures [55]. As an important component of phenotype, the proteome is the ultimate executor of genome function. Proteomics can more directly and accurately reflect the state of cell activity to determine the mechanism of tumor development.

Mitochondrial proteomics is extensively used to identify and quantify proteins in a mitochondrial proteome due to its fast speed and high sensitivity. For example, a total of 5,115 mitochondrial expressed proteins (mtEPs) are identified in mitochondrial samples prepared from human ovarian cancer tissues with iTRAQ (isobaric tags for relative and absolute quantification) [56]. In addition, integrative analysis of mtEPs and transcriptomics data with corresponding clinical characteristics extracted from the TCGA database find that 262 mtEPs are significantly related to the overall survival of ovarian cancer patients. Subsequently, further analysis of those mtEPs identifies several important signaling pathways and candidate biomarkers in ovarian cancers, which might prompt more investigations for new biomarkers [52].

Moreover, Zhan et al. employ iTRAQ-based quantitative proteomics and identify 1,198 mitochondrial differentially expressed proteins (mtDEPs) in human ovarian cancer tissues compared to the controls [57]. Among them, a range of enzymes are enriched in energy metabolism pathways, such as PKM, PFKM, PDHB, CS (citrate synthase), IDH2, isocitrate dehydrogenase 3b (IDH3B), isocitrate dehydrogenase 3a (IDH3A), oxoglutarate dehydrogenase-like (OGDHL), NADH dehydrogenase subunit 5 (ND5), ND2, UQCRH, and CYB. Many of those enzymes could participate in controlling cancer progression. For example, PDHB is the enzyme that catalyzes the conversion of pyruvate to acetyl-CoA, attenuates the production of lactate, and contributes to OXPHOS. PDHB is expressed at low levels in ovarian cancers [58]. CS is overexpressed in ovarian cancers and cell lines compared to controls. In addition, CS can promote cancer cell proliferation, invasion, and migration and constitute a potential therapeutic target in ovarian cancers [59]. Mitochondrial proteomics analysis has found differentially expressed proteins (DEPs) that mainly function in energy metabolism, which may underlie the potential mechanisms of mitochondrial dysfunction [60].

Posttranslational modification (PTM) is usually defined as the enzymatic modification of proteins following protein biosynthesis [61], such as palmitoylation, phosphorylation, ubiquitination, glycosylation, nitration, acetylation, decarboxylation, and methylation [62]. Phosphorylation is one of the common PTMs, which often results in the altered function of a protein.

With the progress of mass spectrometry- (MS-) based proteomics, Li et al. constructed a quantitative mitochondrial phosphoprotein profile of human ovarian cancer [63]. In his study, 124 phosphorylation sites located within 67 mitochondrial phosphoproteins (mtPPs), including 84 phosphorylation sites within 48 mitochondrial differentially phosphorylated proteins (mtDPPs), are identified in ovarian cancer mitochondria compared to controls.

Protein-protein interaction (PPI) network analysis finds that some mtPPs are hub molecules, including cofilin 1 (CFL1), ribosomal protein lateral stalk subunit P2 (RPLP2), eukaryotic translation initiation factor 2 subunit beta (EIF2S2), ribosomal protein lateral stalk subunit P0 (RPLP0), myosin heavy chain 10 (MYH10), voltage-dependent anion channel 3 (VDAC3), thioredoxin-related transmembrane protein 1 (TMX1), heat shock protein family D member 1 (HSPD1), heat shock protein 90 (HSP90), voltage-dependent anion channel 2 (VDAC2), translocase of outer mitochondrial membrane 20 (TOMM20), proteasome 20S subunit alpha 3 (PSMA3), and translocase of outer mitochondrial membrane 20 (TOMM22) [63]. Phosphorylation at a mitochondrial protein is confirmed to possibly modify mitochondrial dysfunction, which offers a fresh understanding of the relationship between mitochondrial protein phosphorylation and potential role in ovarian cancers. For example, phosphorylated cofilin 1 (p-CFL1) is highly expressed in cisplatin-resistant ovarian cancer 317 cell line p-CFL1 and taxol-resistant ovarian cancer cells [64, 65]. In addition, the expression of p-CFL1 in chemoresistant patients is significantly higher than that in chemosensitive patients. PGRMC1 (progesterone receptor membrane component 1) is overexpressed in ovarian cancer cells [66]. Phosphorylation at PGRMC1 probably alters the abundance of mitochondrial proteins and affects mitochondrial content and size [67]. Taken together, these analyses may provide insights into mitochondrial phosphorylation in ovarian cancer.

### 2.4. Metabolomics Study of Mitochondrial Dysfunction Pathways in Ovarian Cancer

In the past 20 years, metabolomics has been continuously developed and served as an essential role in the exploration of pathogenic mechanisms contributing to carcinogenesis, the discovery of novel diagnostic biomarkers, and therapeutic targets. The reprogramming of energy metabolism has been listed as one of the hallmarks of cancer [68]. It is well known that cancer cells undergo metabolic reprogramming to achieve rapid and explosive proliferation [69]. Cancer often causes significant metabolic alterations to sustain its growth, while metabolomics makes early diagnosis of cancer possible.

The metabolic profile of ovarian cancer tissue is significantly different from normal ovarian tissue. One study finds that the differences in metabolite levels are analyzed between serous papillary ovarian cancer (*n* = 44) and healthy controls (*n* = 44) with an ambient ionization technique for MS [70]. These results show that differential metabolites include histamine, purine nucleotide, glycine, serine, and sarcosine. Among them, alanine, serine, cysteine, threonine, and glycine are overexpressed [70]. Another study also finds that metabolic profiling reveals metabolic alterations among ovarian cancers and normal ovaries. Those diversely expressed metabolites include carnitine, acetylcarnitine, butyrylcarnitine, phenylpyruvate, and phenyllactate [71]. Carnitine is a transport protein which transports fatty acids into the mitochondria for *β*-oxidation [72].

Mitochondrial dynamics, also known as mitochondrial morphological alteration, mainly refers to fusion and fission [73–75]. Abnormal mitochondrial dynamics can lead to mitochondrial dysfunction, promote apoptosis, and affect various organ systems, including kidneys, nerves, and cardiovascular system [76–78]. Furthermore, mitochondrial dynamics may also be an important factor inducing metabolic reprogramming in tumors [79]. Mitochondrial elongation factor 2 (MIEF2), located in the mitochondrial outer membrane, serves a regulatory role in mitochondrial fission and is upregulated in ovarian cancer [80]. A metabolomics analysis finds that MIEF2 is overexpressed in ovarian cancer cells to cause an elevated concentration of glyceraldehyde 3-phosphate (GA3P), G6P (glucose 6-phosphate), 3PG (3-phosphoglycerate), F6P (fructose 6-phosphate), and lactate in the glycolytic pathway and a reduced concentration of *α*-ketoglutarate, aconitate, citrate, malate, and fumarate metabolized from the TCA cycle [81]. These results clearly demonstrate that alerted mitochondrial dynamics MIEF2 has a prooncogenic role in ovarian cancers by the shift from OXPHOS to glycolysis.

Consequently, metabolomics is an effective clinical strategy to identify specific biomarkers for 3PM practice in ovarian cancers.

### 2.5. Multiomics Study of Mitochondrial Dysfunction Pathways in Ovarian Cancer

**Figure 2:**
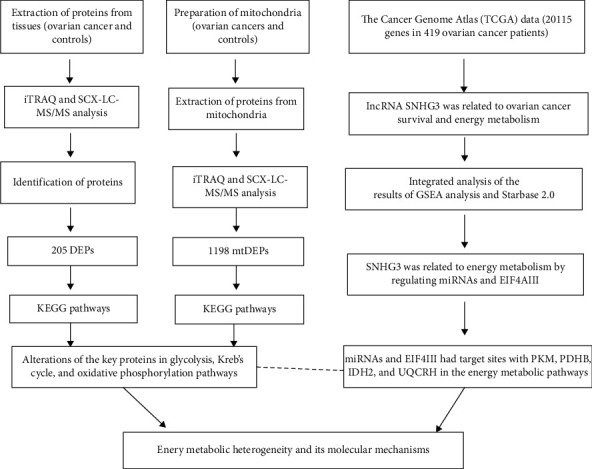
The experimental flow chart of integrative analysis of 1198 mtDEPs, 205 DEPs, and 20115 transcriptomic data from The Cancer Genome Atlas database in ovarian cancers to reveal energy metabolism heterogeneity and its molecular mechanisms. Reproduced from Li et al. [187], with permission from InTech-Open science publisher open access article, copyright 2019. mtDEP: mitochondrial differentially expressed proteins.

The occurrence and development of ovarian cancer is a multifaceted and complex process. Carcinogens induce a wide range of molecular changes at different levels of genome, transcriptome, proteome, and metabolome, and these molecules interact with each other to play critical functions in different cancers [82]. The multiomics approach is an effective strategy to comprehensively study and understand cancer pathogenesis. Li et al. have integrated 1198 mtDEPs derived from mitochondrial proteomics, 205 DEPs from whole-tissue proteomics, and 20115 genes from transcriptomics across 419 ovarian cancer samples in human ovarian cancers [63] (Figure 2), which finds that several energy metabolism-related enzymes are upregulated, including PFKP and PKM in aerobic glycolysis; CS, PDHB, IDH2, and OGDHL in the TCA pathway; and UQCRH in the OXPHOS pathway. In addition, integrative analysis of proteomics and transcriptomics data in ovarian cancers finds that lncRNA SNHG3 is involved in energy metabolism in cancer cells by targeting TCA and OXPHOS pathways [54].

An integrative analysis of proteomics and transcriptomics plus clinical characteristics has demonstrated that lncRNA SNHG3 is able to regulate miRNAs and EIF4AIII that specifically targets the key molecules (PKM, IDH2, PDHB, and UQCRH) in glycolysis, TCA, and OXPHOS pathways. A seven-hub-molecule-signature model (RRAS2, HIST1H2BK, ALB, RPL23A, HIBCH, RPS20, and EIF3E) from those 1198 mtDEPs is established to predict the survival time of ovarian cancer. It clearly demonstrates that mitochondria-based multiomics is an important approach to identifying effective energy metabolism-specific biomarkers and prognostic models for ovarian cancer.

## 3. Interaction of Mitochondrial Dysfunctional Pathway with Oxidative Stress Pathway in Ovarian Cancer

### 3.1. Association of Mitochondrial Dysfunction with Oxidative Stress

Oxidative stress occurs when imbalance occurs between ROS production and antioxidant defenses, which results in oxidation and damage to lipids, proteins, and DNAs. ROS is a collective term for hydrogen peroxide, superoxide anion, and hydroxyl radical [83]. Mitochondrial respiration is the major endogenous source of ROS and produces ~90% ROS [84]. Mitochondria are significantly associated with ROS production, apoptosis, and aging and are susceptible to oxidative stress [85]. Generally, normal cells obtain ROS homoeostasis at a low level of ROS [86]. The surplus ROS initiates lipid peroxidation and causes oxidative damage to mitochondrial membrane systems resulting in depletion of its fluidity and increases Ca^2+^ influx [87]. The dramatic increase in Ca^2+^ influx accelerates the transformation of xanthine dehydrogenase and xanthine oxidase, increases the levels of oxygen free radicals, and activates phospholipase A2 and phospholipase C to degrade a large number of mitochondrial membrane phospholipids, which results in a doubling of arachidonic acid content in the mitochondrial membrane and decreases the function of the electron transport chain, and the damage of the respiratory chain impairs the function of mitochondria to synthesize ATP and impairs OXPHOS, which in turn leads to Ca^2+^ influx and oxygen free radical generation, and forms a “vicious cycle” resulting in mitochondrial respiratory dysfunction and energy metabolism failure [88]. Simultaneously, the increase in Ca^2+^ can aggravate the damage of peroxidation to mitochondria. Decreased membrane potential and membrane fluidity also reduce mitochondrial matrix volume changes and ATP production and cause ion transport dysfunction, intracellular calcium overload, mitochondrial swelling, vacuolization, cristae breakage, and ultimately mitochondrial dysfunction and cell death [89]. Generally, ROS is regarded as a double-edged sword since low levels of ROS can promote tumor cell proliferation, infiltration, metastasis, and chemoradiotherapy resistance [90], whereas excessive levels of ROS over the cytotoxic threshold induce oxidative stress, cause oxidative damage, and initiate apoptotic signaling in cancer cells [91]. Therefore, some anticancer agents can specifically target cancer cells by causing an excessive accumulation of ROS and stimulating ROS-dependent cell death pathways [92].

### 3.2. Different Omics Reveal Mitochondrial Dysfunction and Oxidative Stress Pathways

Continuous progress and innovation in medicine lie in the rapid development of omics technology. Different omics approaches exhibit diverse perspectives to investigate mitochondrial dysfunction and oxidative stress pathways.

A total of 1198 mtDEPs in ovarian cancers relative to controls is identified by iTRAQ-based quantitative proteomics and is further analyzed with Ingenuity Pathway Analysis (IPA) to construct pathway networks and significant signaling pathways [17]. Pathway network analysis reveals the most significant pathways, including mitochondrial dysfunction, antigen presentation, EIF2 signaling, GP6 signaling, glutathione-mediated detoxification, and NRF-mediated oxidative stress response pathways. Recently, metabolic reprogramming has gained significant attention and interest [93]. Cancer cells are able to reprogram their energy metabolism and meet the energy demand. However, current studies on mitochondrial function in cancer energy metabolism have not come to a consistent conclusion. Those mtDEPs enriched in mitochondrial dysfunction pathways are associated with aberrant energy metabolism, including ATP synthase mitochondrial F1 complex assembly factor 1 (ATPAF1), apoptosis-inducing factor mitochondria associated 1 (AIFM1), aconitase 1 (ACO1), copper chaperone for cytochrome c oxidase (COX17), BCL2 apoptosis regulator (BCL2), ATP synthase mitochondrial F1 complex assembly factor 2 (ATPAF2), cytochrome c oxidase subunit 6C (COX6C), cytochrome c oxidase subunit 4I2 (COX4I2), cytochrome c oxidase subunit 4I1 (COX4I1), cytochrome c, somatic (CYCS), cytochrome c oxidase subunit 7A2 like (COX7A2L), cytochrome c oxidase subunit 7A2 (COX7A2), HtrA serine peptidase 2 (HTRA2), glutathione peroxidase 7 (GPX7), thioredoxin 2 (TXN2), monoamine oxidase B (MAOB), and UQCRH. Oxidative stress is a common pathophysiological process in ovarian cancers, which has been proven by numerous multiomics studies in ovarian cancers. Nrf2 (nuclear respiratory factor 2) is an oxidative stress sensor, which plays a core role in oxidative stress response. Nrf2 is modulated by a variety of factors such as ERK5, ERK1/2, Keap1, p38 MAPK, JNK1/2, PI3K/AKT, PKC, and ER stress. Oxidative stress promotes Nrf2 to quickly translocate from cytoplasm to nucleus. Nrf2 induces expressions of many antioxidant genes, ubiquitination/proteasomal degradation proteins, chaperone/stress response genes, phase III detoxifying genes, and phase I and II metabolizing enzymes after Nrf2 binds to antioxidant response element. The mtDEPs enriched in Nrf2-mediated oxidative stress response pathway include actin beta (ACTB), actin alpha cardiac muscle 1 (ACTC1), DnaJ heat shock protein family member B1 (DNAJB1), DnaJ heat shock protein family member B12 (DNAJB12), DnaJ heat shock protein family member C13 (DNAJC3), DnaJ heat shock protein family member C8 (DNAJC8), DnaJ heat shock protein family member C9 (DNAJC9), DnaJ heat shock protein family member C10 (DNAJC10), endoplasmic reticulum protein 29 (ERP29), fibroblast growth factor receptor 4 (FGFR4), flavin containing dimethylaniline monoxygenase 1 (FMO1), ferritin light chain (FTL), glutathione S-transferase alpha 2 (GSTA2), glutathione S-transferase kappa 1 (GSTK1), glutathione S-transferase mu 1 (GSTM1), glutathione S-transferase mu 2 (GSTM2), glutathione S-transferase mu 3 (GSTM3), glutathione S-transferase mu 4 (GSTM4), glutathione S-transferase mu 5 (GSTM5), glutathione S-transferase pi 1 (GSTP1), heme oxygenase 1 (HMOX1), protein kinase C alpha (PRKCA), protein tyrosine phosphatase nonreceptor type 11 (PTPN11), RAS related (RRAS), RAS related 2 (RRAS2), scavenger receptor class B member 1 (SCARB1), and superoxide dismutase 3 (SOD3). Hence, mitochondrial proteomics provides novel insights into the complex molecular pathway underlying ovarian cancer development.

### 3.3. Combination Effects of Mitochondrial Dysfunction and Oxidative Stress in Ovarian Cancer

**Figure 3:**
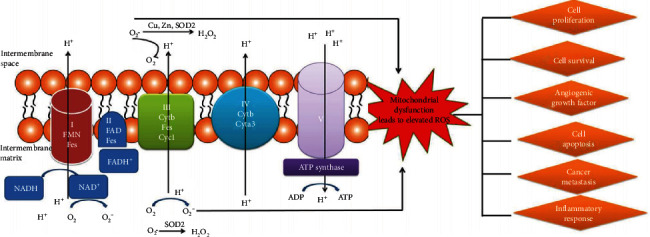
Mitochondrial generation of ROS. Complexes I, II, and III were located on electron transport chain, which plays a pivotal role in the generation of ROS during the process of oxidative phosphorylation. Reproduced from Li and Zhan [188], with permission from *Frontiers* in publisher open access article, copyright 2019.

It is well known that mitochondria are the primary power factories of eukaryotic cells and the primary intracellular source of ROS production. A proteomic study clearly demonstrates that mitochondrial dysfunction and Nrf2-mediated oxidative stress response alterations function in ovarian cancers [17]. Nrf2-mediated oxidative stress response is significantly involved in tumorigenesis and progression of ovarian cancers [94]. Nrf2 is a critical regulator of antioxidant response and is primarily located in the cytoplasm under basal conditions [95]. When the excessive productions of ROS/RNS induce oxidative stress, Nrf2 subsequently translocates to the nucleus and activates the antioxidant response to protect cells against oxidative stress damages [96–99]. Many studies find that ovarian cancer manifests a prooxidant status and is characterized by increased expressions of prooxidant enzymes and decreased expressions of antioxidant enzymes [71, 100–102]. For example, ovarian cancer cells A2780 and OVCAR-3 exhibit higher ROS levels than immortalized ovarian epithelial cells IOSE 397 and IOSE 386 [101]. Mitochondrial dysfunction could lead to elevated levels of ROS in cancer cells, trigger DNA damage through complex biochemical reactions, modulate cancer signaling pathways, and activate oncogenic signals, which promote cancer progression, cancer proliferation, survival, angiogenesis, apoptosis, metastasis, and inflammatory response (Figure 3).

## 4. Mitochondrial Dysfunction Pathway-Based Biomarkers and Therapeutic Targets in Ovarian Cancer

The altered molecules in mitochondrial dysfunction pathways are an important resource to identify specific mitochondria-related biomarkers. These important biomarkers have been identified in ovarian cancer, including superoxide dismutase 2 (SOD2) [103], Sab [104], cytoplasmic estrogen receptor *β*2 (cER*β*2) [105], mimitin [106], TNF receptor-associated protein 1 (TRAP1) [107], mitochondrial ribosomal protein S12 (MRPS12) [108], MIEF2 [81], dynamin-related protein 1 (Drp1) [109], and calcium/calmodulin-dependent protein kinase I (CaMKI) [109]. Current investigations have recognized that the development of cancer is an extremely complex biological process that involves multiple mechanisms and factors, and clinical practice should broadly focus on the comprehensive consideration of multiple factors instead of a one-factor model [110]. An integrated biomarker model based on multiomics has been proposed. Generally, cancer biomarkers can be divided into two main types; type one is used for prediction, diagnosis, and prognosis, and type two is used for exploring underlying mechanisms and therapeutic targets. However, a one-factor model occupies a leading position in published literature. The development of various omics has promoted one to shift from a traditional one-factor model to a multiparameter systematic strategy in the perspective of 3P medicine [82, 110].

In total, 5115 mtEPs in ovarian cancer are identified through iTRAQ-based quantitative mitochondrial proteomics. Further, integrative analysis of these mtEPs and transcriptomics data with clinical information finds that 262 mtEPs are closely linked to the prognosis of ovarian cancers. A total of 63 mtEPs is proposed to serve as candidate biomarkers for ovarian cancer [111]. Those findings are the first reference atlas of human ovarian cancer mitochondrial proteome [56] and are helpful for serving the goal of establishing a human mitochondrial proteomic database and provide an effective clue to discover useful biomarkers for clinical practice [18]. In addition, 102 hub molecules are obtained with a molecular complex detection analysis of 1198 mtDEPs. A seven-hub-molecule-signature model (HIBCH, HIST1H2BK, ALB, EIF3E, RPS20, RRAS2, and RPL23A) from a multivariate regression analysis can predict the survival risks of ovarian cancer [17]. Those mtEPs, mtDEPs, and pattern biomarkers might contribute to the 3PM practice of ovarian cancers after they are further validated with clinical practice.

**Figure 4:**
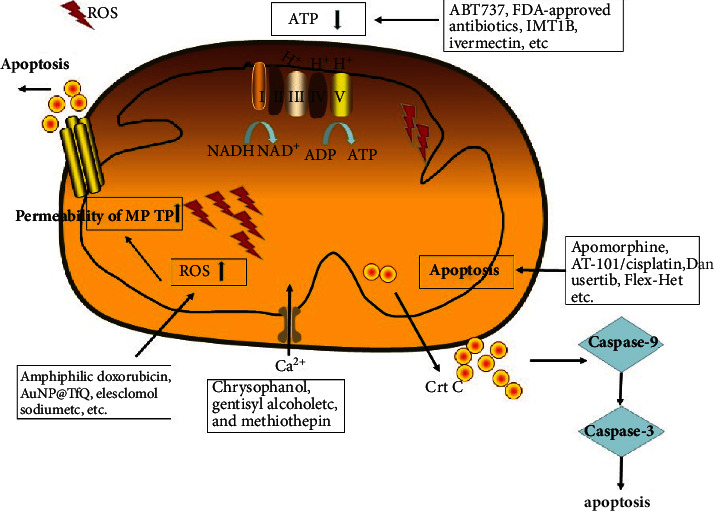
Mechanisms of mitochondrial dysfunction pathway and mitochondria-targeted drugs in ovarian cancer.

Ovarian cancer multiomics-based signaling pathway network studies have found that antigen presentation, mitochondrial dysfunction, EIF2 signaling, GP6 signaling, and glutathione-mediated detoxification pathways might be involved in tumor progression [17]. The mitochondrial dysfunction pathway is a promising target for ovarian cancer therapy, and several potential anticancer agents that block these pathways have been of interest to researchers. Mitochondria-targeting therapy is based on different molecular mechanisms, such as targeting ROS, energy metabolism, permeabilization of mitochondrial outer membrane, permeability of transition pore complex, and photodynamic therapy targeting mitochondria [112]. Numerous studies have revealed that mitochondria-targeting drugs show promising perspectives as potential therapeutics in ovarian cancer clinical practice [113] (Table 1; Figure 4). Mitochondria-targeted therapeutics for ovarian cancer are classified into four broad categories based on different mitochondrial dysfunction pathways: (i) Energy metabolism. The classic anticancer approach targeting the mitochondria usually focuses on function alterations of mitochondrial proteins or changes in energy metabolism. This category of drugs includes bcl-2 inhibitor ABT737 [114], FDA-approved antibiotics [115], IMT1B [116], ivermectin [117], metabolites from invasive Caulerpa species [118], metformin-loaded PLGA-PEG nanoparticles [119], and tigecycline [120]. (ii) Oxidative stress. This category of targeting mitochondrial drugs elevates the levels of ROS, which stimulates the intracytoplasmic release of cytochrome c and triggers apoptosis, for example, amphiphilic doxorubicin [121], AuNP@TfQ [122], elesclomol sodium [123], epoxycytochalasin H [124], eupatilin [125], extract of Persian Gulf Marine Mollusk (Turbo Coronatus) [126], isolinderalactone [127], multifunctional tumor-targeted nanosized ultrasound contrast agents [128], olive leaf extract [129], organoarsenical (PENAO) [130], sideroxylin [131], TEMPO (2,2,6,6-tetramethylpiperidine-1-oxyl) spin label [C_43_H_43_N_6_O_2_Ir_1_·PF_6_]·(Ir·TEMPO1), two TEMPO spin labels [C_52_H_58_N_8_O_4_Ir_1_·PF_6_]˙(Ir-TEMPO2) [132], thiosemicarbazone iron chelators triapine and 2,2′-dipyridyl-N,N-dimethylsemicarbazone [133], and transition metal complexes [Os(*η*^6^-p-cym)(Azpy-NMe_2_)I]^+^(p-cym=p-cymene,Azpy-NMe_2_=2-(p-[dimethylamino] phenylazo)pyridine) [134]. (iii) Cell apoptosis. Activation of apoptotic pathways is a predominant antitumor mechanism. This category of drugs includes a hybrid drug dichloroacetate-platinum(II) [DCA-Pt(II)] [135], a monocationic, square-planar platinum(II) complex [Pt(BDI(QQ))] [136], apomorphine [137], AT-101/cisplatin [138], chaetomugilin J [139], danusertib [140], epothilones [141], exosomal microRNAs [142], Flex-Het [143], gedunin isolated [144], gentiopicroside [145], glycyrrhetinic acid rhodamine B benzyl amide 35 [146], hedyotis diffusa willd [147], jaceosidin [148], lytic peptides (YX-1) [149], modified mitochondria-targeted chlorambucil compounds [150], niclosamide [151], nilotinib [152], piperine [153], poly(lactic-co-glycolic acid) (PLGA) nanoparticles (NPs-cRGD) [154], Polyphyllin VII [155], quercetin [156], rGO-Ag [157], RY-2f [158], sambucus nigra agglutinin [159], spiropyrazoline oxindoles compound 1a [160], STX140 [161], STX641 [161], SW III-123 [162], swerchirin [163], Z-Ligustilidein [164], *α*-mangostin [165], BCL-2/XL inhibition (ABT-263) [166], shikonin [167], and 3,7,14,15-tetraacetyl-5-propanoyl-13(17)-epoxy-8,10(18)-myrsinadiene (TPEM) [168]. (iv) Calcium overload and autophagy. This category of drugs includes chrysophanol [169], gentisyl alcohol [170], methiothepin [171], and BMI1 inhibitor (PTC-209) [172]. Interestingly, several FDA-approved antibiotics are found to target mitochondrial OXPHOS and inhibit ovarian cancer cell lines. Meanwhile, ivermectin, a drug for malaria, could repress ovarian cancer cells through targeting energy metabolism. These findings suggest the progress of identifying new use of existing drugs is helpful in diminishing the medical burden and reducing the cost of drugs.

## 5. Future Perspective

The present review has comprehensively discussed the roles and contributions of integrative omics/multiomics in clarification of mitochondrial dysfunction pathways of ovarian cancers, establishment of multiomics-based signaling pathway networks, pattern biomarkers, and therapeutic targets for effective 3P medicine of ovarian cancers. Moreover, we would like to highlight and discuss several future directions for research and practice in ovarian cancers.

### 5.1. Expanded Multiomics Study of Mitochondrial Dysfunction Pathway in Ovarian Cancer

As the rapid development of omics technologies is evolved in, the research scope is not only limited to genomics, transcriptomics, proteomics, and metabolomics but also expanded to other multiomics approaches such as lipidomics, glycomics, and radiomics. The main objective of lipidomics is to identify and quantify cellular lipid molecular species and their function in biological progress [173, 174]. The lipid molecules in mitochondria exert diverse biological functions in the activity of the electron transport chain, the production of ROS, and mitochondrial dynamics [175, 176]. With these features, mitochondrial lipidome has tremendous potential to considerably enrich our understanding of the mitochondrial dysfunction pathway.

As the main executive substance of life function, the functional study of protein has been favored. One of the main reasons why proteins are versatile is PTMs. This is because after individual amino acid residues in a protein are covalently modified, their physical and chemical properties, conformation, etc., are also changed, which can significantly affect the functions of the protein. PTM is a process that occurs after the completion of protein translation and covalently binds various types of chemical small molecule groups or small proteins to specific amino acid residues in the substrate. At present, there are as many as hundreds of known PTMs, and PTMs occur in almost all proteins, and the same protein also has a variety of PTMs at the same time, which can not only exponentially expand the number of proteoforms but also endow more complexity with human life processes. A variety of PTMs has been identified, including glycosylation, acetylation, methylation, phosphorylation, and ubiquitination [177]. Protein glycosylation, which is a common form of PTMs, refers to the addition of a multitude of glycans to peptides [178]. This modification occurs primarily to alter the structural conformation and regulate protein interactions, activity, localization, and stability [179]. Abnormal glycosylation has been related to a variety of human diseases, such as cancer and neurodegenerative, and cardiovascular diseases [180]. One MS-based glycoproteomic characterization of 119 TCGA high-grade serous ovarian cancer (HGSOC) tissues classifies HGSOC into 3 major tumor clusters and 5 groups of glycopeptides. Different tumor subtypes show different glycopeptide combination preferences, and intact glycopeptide (IGP) 3/4/1 glycopeptides are highly expressed in subtypes 1/2/3, respectively. IGP1 mainly contains fucose glycans, IGP3 contains fucose and sialic acid residue glycans, and IGP4 mainly contains high mannose glycans [181]. In addition, acetylation, another PTM, occurs mainly on chromatin histones and maintains a dynamic balance under the action of histone acetyltransferases with deacetyltransferases. Protein acetylation is a key PTM in cellular regulation. In general, acetylation can loosen the nucleosome structure, facilitate the binding of transcriptional factors to gene promoter regions, and activate gene transcription [182]. Mitochondrial lysine acetylation is found to initiate mitochondrial degradation by autophagy [183]. Protein phosphorylation is the most common PTM in eukaryotes. Phosphorylation strongly influences many cellular events, such as transcriptional activity, protein subcellular localization, protein stability, and enzymatic activity [184]. Recently, one study indicates that phosphorylation at residue S616 in mitochondrial Drp1 triggers autophagy for chemoresistance and regrowth in the surviving cancer cells [185]. Ubiquitination refers to that ubiquitin molecules covalently bind to target proteins under the action of E1 activating enzyme, E2 coupling enzyme, and E3 ligase. The ubiquitin-labeled target proteins are recognized and degraded by 26s proteasome, which can remove wrong proteins and regulate the cell cycle and immune function of the body. Parkin mediates ubiquitination of mitochondrial outer membrane proteins including TOMM20, mitofusin, mitochondrial Rho (miro), and VDAC, forming a recognition site for adaptor proteins, and promoting the selective autophagy of damaged mitochondria [186]. Future studies are needed to further expand multiomics study and integrate data to obtain a multiomics understanding of the mitochondrial dysfunction pathway in ovarian cancer.

### 5.2. Development of Mitochondrial Dysfunctional Pathway-Based Biomarker and Therapeutic Targets in Ovarian Cancer for 3P Medicine Practice in Ovarian Cancer

The complex molecular interaction networks constructed from mitochondrial dysfunctional pathways based on multiomics data could provide new perspectives to elucidate molecular mechanisms, discover novel therapeutic targets, and identify effective and reliable pattern biomarkers for 3PM practice in ovarian cancers [62]. Some mitochondrial dysfunctional pathway-based pattern biomarkers and multiple therapeutic targets have been discovered. However, those studies are not satisfactory enough in ovarian cancers due to the following factors: (i) Omics studies of mitochondrial protein PTMs are still in the exploratory stage. To date, no studies on mitochondrial protein PTMs in ovarian cancers have been reported, with the exception of mitochondrial phosphoproteome. (ii) Metabolites have not been completely investigated. (iii) The existing database limits the ability to discover tumor heterogeneity. Most of the existing databases contain different information of high-grade serous ovarian cancer (HGSOC), lacking other histological types of ovarian cancers. (iv) The large-sample size studies are needed to verify mitochondrial dysfunctional pathway-based pattern biomarkers in clinical practice. As a consequence, it is necessary to widen the scope of omics research to maximize the coverage of genome, transcriptome, proteome, and metabolome.

### 5.3. Phenome-Centered Mitochondrial Dysfunctional Pathway Study in Ovarian Cancer

**Figure 5:**
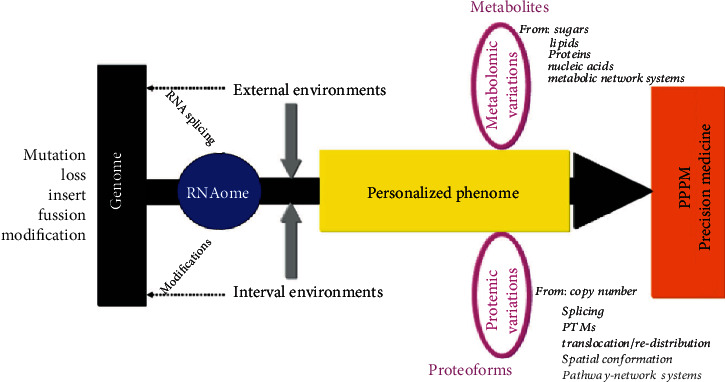
Phenome-centered multiomics studies in ovarian cancers. The center of multiomics is being moved from genomics to phenomics, especially proteomics and metabolomics. Modified from Zhan et al. [189], with permission from Elsevier publisher, copyright 2018, and reproduced from Li et al. [62], with permission from Wiley publisher, copyright 2021.

With the advancement of high-throughput technologies, the core of multiomics is shifting from genomics to phenomics (Figure 5). The term “phenomics,” first presented by Dr. Steven A. Garan in 1996, refers to the discipline of systematically studying all phenotypes of a certain organism or cell under the complex conditions. The phenome is a range of measurable features, including physical, chemical, and radiographic features of individuals and populations, which are derived from the complex interactions of genes, epigenetics, proteins, and metabolites. Proteomics and metabolomics are the two major components of phenomics. Protein is one of the important structural and functional units of life activities. Proteomics essentially refers to the protein features at the large-scale level, including protein expression, PTMs, and protein-protein interactions, from which there is a global understanding of the initiation and progression of diseases on the protein level. Metabolomics, with the goal of identification and quantification of all endogenous and exogenous metabolites in tissues, cells, and biofluids, could reflect changes in biological phenotypes more directly and immediately. Phenome-centered multiomics study will be a long-term trend for future cancer studies.

## 6. Conclusions

Mitochondrial dysfunctional pathway alterations are the important hallmarks of ovarian cancer. Great progress has been achieved to understand mitochondrial dysfunction pathway alterations in ovarian cancer. However, molecular mechanisms and their regulation of mitochondrial dysfunctional pathways in ovarian cancer remain unknown. The published evidence demonstrates that mitochondrial dysfunction is crosslinked with various complex biological processes, including energy metabolism, oxidative stress, cell apoptosis, cell cycle, mitophagy, and immunity process. This review provides the evidence base for the comprehensive understanding of underlying mechanisms between mitochondrial dysfunction and tumorigenesis in ovarian cancer. It holds the promise to develop new candidate biomarkers and therapeutic targets based on mitochondrial dysfunction pathways to effectively treat ovarian cancer in the context of 3P medicine.

## Figures and Tables

**Table 1:** Some drugs targeting mitochondrial dysfunction as a therapeutic strategy for ovarian cancer treatment.

Mitochondrial dysfunction pathway	Drugs	Functional mechanism	Cell model	References
Energy metabolism	Bcl-2 inhibitor ABT737	Disrupting Bcl2-dependent OXPHOS	SKOV3 (cisplatin-sensitive) and SKOV3/DDP (cisplatin-resistant)	[114]
	FDA-approved antibiotics	Inhibit mitochondrial OXPHOS and/or biogenesis	Ovarian cancer stem cells	[115]
	IMT1B	Targeting the human mitochondrial RNA polymerase and affecting the biogenesis of OXPHOS	A2780 cells	[116]
	Ivermectin	Targeted lncRNA-EIF4A3-mRNA pathways	A2780 and TOV-21G cells	[117]
	Metabolites from invasive Caulerpa species	Selectively inhibiting respiratory complex II activity	OV2008 and C13 cells	[118]
	Metformin-loaded PLGA-PEG nanoparticles	OXPHOS	SKOV-3 ovarian cells	[119]
	Tigecycline	Inhibiting translation by mitochondrial ribosome	SW626 and SKOV-3 cells	[120]

Oxidative stress	Amphiphilic doxorubicin	Tumor- and mitochondria-selective deliveries of the anticancer drug	The OVCAR-8 human ovarian carcinoma cell line and its doxorubicin-resistant derivative NCI/ADR-RES cell line	[121]
		Induces an increased level of ROS generation	PA-1 cells	[122]
	Elesclomol sodium	Accumulation of ROS	RMG1 and OVCA432 cells	[123]
	Epoxycytochalasin H	Increased ROS level in cells	A2780 cells	[124]
	Eupatilin	Reactive oxygen species (ROS) generation, calcium influx	OV90 and ES2 cells	[125]
	Extract of Persian Gulf Marine Mollusk (Turbo Coronatus)	ROS-mediated mitochondrial targeting	Human epithelial ovarian cancer cells	[126]
	Isolinderalactone	Mitochondrial superoxide-mediated pathways	SKOV-3 and OVCAR-3 cells	[127]
	Multifunctional tumor-targeted nanosized ultrasound contrast agents	Consumed GSH and enhanced reactive ROS level	A2780 and SKOV-3 cells	[128]
	Olive leaf extract	Increasing ROS and decreasing activity of ROS scavenging enzymes	OVCAR-3 cells	[129]
	Organoarsenical (PENAO)	ROS production and mitochondrial depolarization	OVCAR-3, SKOV-3, TOV112D, TOV21G, and EFO27 cells	[130]
	Sideroxylin	The induction of mitochondrial dysfunction and the activation of PI3K and MAPK signal transduction	ES2 and OV90 cells	[131]
	TEMPO (2,2,6,6-tetramethylpiperidine-1-oxyl) spin label [C_43_H_43_N_6_O_2_Ir_1_·PF_6_]˙ (Ir-TEMPO1) and two TEMPO spin labels [C_52_H_58_N_8_O_4_Ir_1_·PF_6_]˙ (Ir-TEMPO2)	Antiproliferative activity and antioxidant activity	A2780 cells	[132]
	Thiosemicarbazone iron chelators triapine and 2,2′-dipyridyl-N,N-dimethylsemicarbazone	Promote selective oxidation of mitochondrial Prx3	A2780 and OVCAR-3 cells	[133]
	Transition metal complexes [Os(*η*^6^-p-cym)(Azpy-NMe_2_)I]^+^(p-cym=p-cymene, Azpy-NMe_2_=2-(p-[dimethylamino] phenylazo)pyridine)	Superoxide formed in the first step of O_2_ reduction in mitochondria	A2780 cells	[134]

Cell apoptosis	A hybrid drug dichloroacetate-platinum(II) [DCA-Pt(II)]	Mitochondria-mediated apoptosis	A2780 and A2780DDP cells	[135]
	A monocationic, square-planar-platinum(II) complex [Pt(BDI(QQ))]	Induces DNA damage, leading to p53 enrichment, mitochondrial membrane potential depolarization, and caspase-mediated apoptosis	A2780 cells	[136]
	Apomorphine	Inducing caspase activation and mitochondrion-associated apoptosis	ES2 and OV90 cells	[137]
	AT-101/cisplatin	Reducing some pivotal antiapoptotic proteins such as Bcl-2, HIF-1A, cIAP-1, and XIAP	OVCAR-3 cells	[138]
	Chaetomugilin J	Enhancing apoptosis through inhibiting Pink1/Parkin-mediated mitophagy	A2780 cells	[139]
	Danusertib	Danu induced mitochondria-dependent apoptosis and autophagy in a dose- and time-dependent manner	C13 and A2780cp cells	[140]
	Epothilones	The induction of apoptosis via mitochondrial pathway	OV-90 cells	[141]
	Exosomal microRNAs	Inhibitory effects on cells by blocking the cell cycle and activating mitochondria-mediated apoptosis signaling	A2780 and SKOV-3 cells	[142]
	Flex-Het	Promoting mitochondrial-mediated apoptosis	A2780 and OVCAR-3 cells	[143]
	Gedunin isolated	Triggered severe ROS generation leading to DNA damage and cell cycle arrest in G2/M phase thus inhibiting cell proliferation. ROS upregulation also led to mitochondrial stress and membrane depolarization, which eventually resulted in mitochondria-mediated apoptosis following cytochrome C release, caspase 9 and 3 activation, and PARP cleavage	PA-1 and OVCAR-3 cells	[144]
	Gentiopicroside	Loss of MMP and induction of apoptosis	SKOV-3 cells	[145]
	Glycyrrhetinic acid rhodamine B benzyl amide 35	Triggered apoptosis	A2780 cells	[146]
	Hedyotis diffusa willd	Induced through the mitochondria-associated apoptotic pathway	A2780 cells	[147]
	Jaceosidin	Induction of apoptosis involving cytochrome c release from mitochondria to cytosol	CAOV-3 cells	[148]
	Lytic peptides (YX-1)	Activating the mitochondria apoptotic pathway	A2780 cells	[149]
	Modified mitochondria-targeted chlorambucil compounds	Effectively evading multidrug resistance resulting from cytosolic GST-*μ* upregulation by rapid E28 accumulation in mitochondria	A2780 cells	[150]
	Niclosamide	Inducing mitochondrial uncoupling	HCT116 p53+/+(H2B-GFP) and HCT116 p53−/−(H2B-RFP) ovarian cell lines	[151]
	Nilotinib	Reducing the viability of human ovarian cancer cells via mitochondrion-mediated apoptosis	SKOV-3 cells	[152]
	Piperine	Release of mitochondrial cytochrome c to cytosol, activation of caspases 3 and 9	A2780 cells	[153]
	Poly(lactic-co-glycolic acid) (PLGA) nanoparticles (NPs-cRGD)	Upregulated the expression of p53 and promoted high levels of reactive oxygen species to induce the mitochondrial apoptosis pathway	SKOV3 and SKOV3-DDP cells	[154]
	Polyphyllin VII	Induces mitochondrial apoptosis by regulating the PP2A/AKT/DRP1 signaling axis	A2780 and SKOV-3 cells	[155]
	Quercetin	Induces mitochondrial-mediated apoptotic pathway	PA-1 cells	[156]
	rGO-Ag	Reducing cell viability by mediating the generation of ROS, leaking lactate dehydrogenase, reducing mitochondrial membrane potential, and enhancing expression of apoptotic genes	A2780 cells	[157]
	RY-2f	Upregulation of p21, cyclin B1, Bax, Bad and cleaved-PARP, and suppression of cyclin A, CDK2, and Bcl-2	A2780/CDDP cells	[158]
	Sambucus nigra agglutinin	Inducing apoptosis in ovarian cancer cells	OAW-42 and SKOV-3 cells	[159]
	Spiropyrazoline oxindoles compound 1a	Mitochondria-mediated apoptosis and autophagy	A2780 cells	[160]
	STX140 and STX641	Depolarizing mitochondrial bioenergetics and activate caspase 3/7	A2780 cells	[161]
	SW III-123	Activated both intrinsic and extrinsic apoptotic pathways	SKOV-3 cells	[162]
	Swerchirin	Induction of mitochondrial apoptosis	SKOV-3 cells	[163]
	Z-Ligustilidein	Triggering oxidative stress and inducing apoptosis	OVCAR-3 cells	[164]
	*α*-Mangostin BCL-2/XL inhibition (ABT-263) Shikonin	The involvement of reactive oxygen species and mitochondrial-mediated apoptosis Apoptosis regulated by BCL2-family proteins Inducing mitochondria-mediated apoptosis	OVACAR-3 cells OVCAR3, OV90, OAW28, OVCAR8, OVSAHO, OVCAR5, TKYNU, JHOM1, and RMUGS cells A2780-CR cells	[165] [166] [167]
	3,7,14,15-Tetra-acetyl-5-propanoyl-13(17)-epoxy-8,10(18)-myrsinadiene (TPEM)	Promoting mitochondrial-mediated apoptosis	OVCAR-3 and Caov-4 cells	[168]
	Chrysophanol	Inducing cell death and inhibiting invasiveness via mitochondrial calcium overload	ES2 and OVCAR3 cells	[169]

Calcium overload	Gentisyl alcohol	Loss of mitochondrial membrane potential with calcium dysregulation	ES2 and OV90 cells	[170]
	Methiothepin	Epolarization of the mitochondrial membrane and increased mitochondrion-specific Ca2+ levels and decreased ATP production and oxidative phosphorylation	ES2 and OV90 cells	[171]

Autophagy	BMI1 inhibitor (PTC-209)	Targeting PINK1-PARK2-dependent mitochondrial pathway	OVCAR4 and CP20 cells	[172]

## Data Availability

All data are present in the table and figures in this article; they can be available from the journal website.

## Abbreviations

ACO1: Aconitase 1
ACO2: Aconitase 2
ACTB: Actin beta
ACTC1: Actin alpha cardiac muscle 1
AIFM1: Apoptosis-inducing factor mitochondria associated 1
ALB: Albumin
ATPs: Adenosine triphosphate
ATP5B: ATP synthase F1 *β* subunit
ATPAF1: ATP synthase mitochondrial F1 complex assembly factor 1
ATPAF2: ATP synthase mitochondrial F1 complex assembly factor 2
BCL2: BCL2 apoptosis regulator
CAMK2D: Calcium/calmodulin-dependent protein kinase D
CaMKI: Calcium/calmodulin-dependent protein kinase I
cER*β*2: Cytoplasmic estrogen receptor *β*2
CO1: Cytochrome c oxidase 1
COX17: Copper chaperone for cytochrome c oxidase
COX4I2: Cytochrome c oxidase subunit 4I2
COX4I1: Cytochrome c oxidase subunit 4I1
COX6C: Cytochrome c oxidase subunit 6C
COX7A2: Cytochrome c oxidase subunit 7A2
COX7A2L: Cytochrome c oxidase subunit 7A2 like
CS: Citrate synthase
CYCS: Cytochrome c, somatic
D-loop: Displacement loop
DNAJB1: DnaJ heat shock protein family member B1
DNAJB12: DnaJ heat shock protein family member B12
DNAJC3: DnaJ heat shock protein family member C13
DNAJC8: DnaJ heat shock protein family member C8
DNAJC9: DnaJ heat shock protein family member C9
DNAJC10: DnaJ heat shock protein family member C10
Drp1: Dynamin-related protein 1
EIF2: Eukaryotic translation initiation factor 2
EIF2S2: Eukaryotic translation initiation factor 2 subunit beta
EIF3E: Eukaryotic translation initiation factor 3 subunit E
ENO1: Enolase 1
ER: Endoplasmic reticulum
ERK5: Extracellular signal-regulated kinase 5
ERK1/2: Extracellular signal-regulated kinase 1/2
ERP29: Endoplasmic reticulum protein 29
ESRRA: Estrogen-related receptor alpha
FGFR4: Fibroblast growth factor receptor 4
FMO1: Flavin containing dimethylaniline monoxygenase 1
FTL: Ferritin light chain
F6P: Fructose 6-phosphate
GA3P: Glyceraldehyde 3-phosphate
G6P: Glucose 6-phosphate
GEO: Gene Expression Omnibus
GLUT1: Glucose transporter 1
GP6: Glycoprotein VI platelet
GPI: Glycosylphosphatidylinositol
GPX7: Glutathione peroxidase 7
GSTA2: Glutathione S-transferase alpha 2
GSTK1: Glutathione S-transferase kappa 1
GSTM1: Glutathione S-transferase mu 1
GSTM2: Glutathione S-transferase mu 2
GSTM3: Glutathione S-transferase mu 3
GSTM4: Glutathione S-transferase mu 4
GSTM5: Glutathione S-transferase mu 5
GSTP1: Glutathione S-transferase pi 1
HIF1: Hypoxia-inducible factor 1
HIBCH: 3-Hydroxyisobutyryl-CoA hydrolase
HIST1H2BK: Histone cluster 1, H2bk
HK2: Hexokinase 2
HGSOC: High-grade serous ovarian cancer
HMOX: Heme oxygenase 1
HSPD1: Heat shock protein family D member 1
HSP90: Heat shock protein 90
HTRA2: HtrA serine peptidase 2
iTRAQ: Isobaric tags for relative and absolute quantification
IDH2: Isocitrate dehydrogenase 2
IDH3A: Isocitrate dehydrogenase 3a
IDH3B: Isocitrate dehydrogenase 3b
IGP: Intact glycopeptide
JNK1/2: cJun N-terminal kinases 1/2
Keap1: Kelch-like ECH associated protein 1
LDHA: Lactate dehydrogenase A
lncRNA: Long noncoding RNA
MAOB: Monoamine oxidase B
MAPK: Mitogen-activated protein kinase
MIEF2: Mitochondrial elongation factor 2
Miro: Mitochondrial Rho
MRPS12: Mitochondrial ribosomal protein S12
MTERF: Mitochondrial transcription termination factor
MYH10: Myosin heavy chain 10
mtDNA: Mitochondrial DNA
mtDEP: Mitochondrial differentially expressed protein
mtDPP: Mitochondrial differentially phosphorylated protein
mtEP: Mitochondrial expressed protein
mTOR: Mechanistic target of rapamycin
nDNA: Nuclear DNA
ND2: NADH dehydrogenase subunit 2
ND5: NADH dehydrogenase subunit 5
Nrf1: Nuclear factor erythroid-derived 2-related factor 1
Nrf2: Nuclear respiratory factor 2
OGDHL: Oxoglutarate dehydrogenase-like
OXPHOS: Oxidative phosphorylation
PARP: Poly (ADP-ribose) polymerase
PDHB: Pyruvate dehydrogenase E1 component subunit *β*
PDK1: Protein kinase-1
PFKM: Phosphofructokinase
PGRMC1: Progesterone receptor membrane component 1
PI3K: Phosphatidylinositol 3-kinase
PKC: Protein kinase C
PKM: Pyruvate kinase
PKM2: Pyruvate kinase 2
PPARGC1A: Peroxisome proliferator-activated receptor gamma, coactivator 1 alpha
PRKCA: Protein kinase C alpha
PTPN11: Protein tyrosine phosphatase nonreceptor type 11
PTMs: Posttranslational modifications
PSMA3: Proteasome 20S subunit alpha 3
RRAS: RAS related
RRAS2: RAS related 2
ROS: Reactive oxygen species
RNS: Reactive nitrogen species
RPLP2: Ribosomal protein lateral stalk subunit P2
RPLP0: Ribosomal protein lateral stalk subunit P0
RPL23A: Ribosomal protein L23a
RPS20: Ribosomal protein S20
SCARB1: Scavenger receptor class B member 1
SOD2: Superoxide dismutase 2
SOD3: Superoxide dismutase 3
TCGA: The Cancer Genome Atlas
TMX1: Thioredoxin-related transmembrane protein 1
TIMMDC1: Translocase of inner mitochondrial membrane domain-containing protein 1
TOMM20: Translocase of outer mitochondrial membrane 20
TOMM22: Translocase of outer mitochondrial membrane 22
TME: Tumor microenvironment
TRAP1: TNF receptor-associated protein 1
TXN2: Thioredoxin 2
UQCRH: Ubiquinol-cytochrome c reductase hinge protein
VDAC2: Voltage-dependent anion channel 2
VDAC3: Voltage-dependent anion channel 3
3P medicine: Predictive, preventive, and personalized medicine
3PG: 3-Phosphoglycerate.
